# Distinct transcriptional, cytokine, and γ-protocadherin responses to TLR1/2, TLR3, and TLR7/8 agonists in human cardiac endothelial cells

**DOI:** 10.1371/journal.pone.0361988

**Published:** 2026-11-12

**Authors:** Aditi Chaudhari, Fredrika Gustafsson, Lillemor Mattsson Hultén, Victoria Rotter Sopasakis

**Affiliations:** 1 Department of Laboratory Medicine, Institute of Biomedicine, Sahlgrenska Academy, University of Gothenburg, Gothenburg, Sweden; 2 Department of Clinical Chemistry, Sahlgrenska University Hospital, Gothenburg, Sweden; 3 Wallenberg Laboratory, Department of Molecular and Clinical Medicine, Institute of Medicine, Sahlgrenska Academy, University of Gothenburg, Gothenburg, Sweden; Bangladesh Agricultural University, BANGLADESH

## Abstract

Toll-like receptors (TLRs) are key mediators of innate immune signaling and have been implicated in cardiovascular disease. TLR1, TLR3, and TLR7 are upregulated in diseased myocardium, but responses to ligands engaging these receptors in cardiac endothelial cells (ECs) remain poorly characterized. In the present study, primary human cardiac microvascular ECs were stimulated for 24 hours with Pam3CSK4, a TLR1/TLR2 agonist, Poly I:C, a TLR3 agonist, or R848, a TLR7/TLR8 agonist. Basal and stimulus-induced expression of TLR1, TLR2, TLR3, TLR7, and TLR8 was assessed by RT-qPCR. In addition, expression of a predefined panel of 84 NF-κB pathway-associated genes was profiled, secreted cytokine concentrations were quantified, and γ-protocadherin (PCDHG) family transcript levels were determined. TLR1, TLR2, and TLR3 were readily detectable in untreated ECs, whereas TLR7 and TLR8 transcripts were undetectable. Poly I:C markedly increased expression of TLR2, TLR3, and TLR7, while Pam3CSK4 and R848 did not significantly alter expression of the TLRs examined. The three stimuli produced distinct transcriptional profiles within the focused gene panel. Poly I:C induced extensive transcriptional changes and the strongest cytokine response while reducing all detectable PCDHG transcripts. Pam3CSK4 also induced extensive transcriptional changes and cytokine secretion but generated a different expression profile and generally increased PCDHG transcript levels. R848 produced limited transcriptional changes and did not significantly alter cytokine secretion or PCDHG expression at the 24-hour time point. These findings demonstrate stimulus-dependent differences in inflammatory and PCDHG responses in cardiac ECs and show that the endothelial TLR repertoire itself is differentially regulated by innate immune stimulation. Under the conditions tested, Poly I:C elicited the strongest inflammatory response and the most pronounced reduction in PCDHG expression, whereas R848 produced comparatively limited effects. Differences from previously observed responses in cardiac fibroblasts further highlight the cell-type-specific nature of innate immune responses in the human heart.

## Introduction

Endothelial cells (ECs) constitute a substantial proportion of the cellular composition of the adult human heart, accounting for approximately 8–12% of total cardiac cells, with regional variation between atrial and ventricular tissue. Within the non-cardiomyocyte compartment, ECs comprise approximately 16% of all cells, as demonstrated by recent single-nucleus transcriptomic analyses [1].

Consequently, cardiac ECs are integral to heart function, forming the inner lining of blood vessels and regulating vascular tone and blood flow. Their dysfunction is closely linked to various heart diseases, including atherosclerosis, hypertension, coronary artery disease and heart failure [2–4]. Cardiac ECs and cardiomyocytes (CMs) maintain essential, reciprocal communication that supports heart function [5]. ECs release paracrine signals—such as nitric oxide (NO), endothelin‑1, neuregulin‑1 (NRG‑1), and apelin—that directly modulate CM contractility, growth, survival, and development [6]. Impairment in this signaling—often due to endothelial dysfunction—can contribute to pathological remodeling and reduced cardiac output. Simultaneously, ECs and fibroblasts engage in complex crosstalk via paracrine factors, direct cell–cell contacts, and extracellular matrix (ECM) remodeling [7]. These interactions are critical for tissue repair and homeostasis post-injury; dysfunction in these processes can exacerbate fibrosis and deteriorate heart performance.

Beyond their structural and regulatory roles in the cardiovascular system, endothelial cells are also active participants in immune surveillance. Positioned at the interface between the vessel wall and circulating blood, ECs encounter both microbial products and endogenous molecules released during tissue injury. Their expression of Toll-like receptors (TLRs) enables them to recognize pathogen-associated molecular patterns and damage-associated molecular patterns and to initiate innate immune responses. In ECs, TLR stimulation can influence cytokine and chemokine production, vascular tone, permeability, and leukocyte trafficking, thereby linking immune activation to vascular function (reviewed in [8]). These responses may also affect endothelial cell–cell interactions and signaling networks that are essential for maintaining microvascular integrity.

Molecules that regulate endothelial cell–cell communication are therefore of particular interest in the context of TLR-induced inflammation. Protocadherins constitute a large and structurally diverse subgroup of the cadherin superfamily [9,10]. In addition to participating in calcium-dependent cell–cell interactions, protocadherins have been implicated in intracellular signaling, tissue organization, and regulation of gene expression [11,12]. Although they have been studied primarily in the nervous system, several protocadherins, including members of the γ-protocadherin family, are expressed in endothelial cells [13]. Studies in brain microvascular and aortic ECs suggest that individual γ-protocadherins can influence endothelial junction-related processes, inflammatory responsiveness, and signaling pathways involved in vascular homeostasis [14,15]. However, their effects appear to differ among individual family members and vascular beds, and little is known about their regulation or function in human cardiac microvascular ECs.

The possible relationship between TLR-mediated inflammation and γ-protocadherin regulation emerged from our previous studies of human myocardial tissue. We found increased expression of TLR1, TLR3, and TLR7 and several associated inflammatory mediators in myocardium from patients with advanced coronary artery disease or aortic valve disease [16]. In a subsequent transcriptomic study of myocardial tissue from comparable patient groups, we observed pronounced reductions in several γ-protocadherin transcripts alongside a broadly increased inflammatory state [17]. More recently, we investigated this potential connection in human cardiac fibroblasts and found that Pam3CSK4 (a TLR1/TLR2 stimulator), Poly I:C (a TLR3 stimulator), and R848 (a TLR7/TLR8 stimulator), induced different transcriptional and cytokine responses and differentially regulated PCDHG expression, with Poly I:C producing the most consistent reduction in PCDHG transcript levels [18]. Whether cardiac ECs exhibit a similar association between innate immune stimulation and PCDHG regulation, or whether these responses are specific to cardiac fibroblasts, remains unknown.

Accordingly, the present study examined the responses of primary human cardiac microvascular ECs to Pam3CSK4, Poly I:C, and R848. We assessed changes in the expression of a predefined panel of NF-κB pathway-associated genes, secretion of proinflammatory cytokines, and PCDHG transcript abundance. By characterizing these responses in cardiac ECs and considering them in relation to our previous findings in cardiac fibroblasts, we aimed to identify shared and endothelial-specific features of innate immune and PCDHG regulation in the human heart.

## Materials and methods

### Cell culture

Primary human cardiac microvascular endothelial cells from a 34 year old female donor (cat# CC-7030, Lonza Inc, Basel, Switzerland) were seeded at a density of 7,000 cells/cm^2^ in 6-well TPP culture plates and maintained in Microvascular Endothelial Growth Medium 2 (cat# CC-3202, Lonza Inc, Basel, Switzerland). Confluent cultures were exposed for 24 hours to one of the following agonists or to vehicle alone: 100 ng/ml Pam3CysSerLys4 (Pam3CSK4; TLR1/TLR2 heterodimer agonist, cat# NBP2-25297, Novus Biologicals, Centennial, CO), 100 µg/ml polyinosinic:polycytidylic acid (Poly I:C; TLR3 agonist, cat# NBP2-25288, Novus Biologicals, Centennial, CO), 5 µg/ml R848 (TLR7/TLR8 agonist, cat# NBP2-26231, Novus Biologicals, Centennial, CO), or 10 ng/ml lipopolysaccharide (LPS) from E. coli (Invivogen, San Diego, CA). Agonist concentrations were selected on the basis of maximal receptor activation as established by the manufacturers and confirmed in the literature [19,20]. Dimethyl sulfoxide (DMSO; Sigma-Aldrich, Saint Louis, MO) served as the vehicle control. All cell-culture experiments were performed in two independent experimental runs, with four parallel culture replicates per treatment condition in each experiment (total n = 8 culture replicates per condition).

### RNA extraction and gene expression analysis

Cells were rinsed twice with ice-cold PBS containing Ca^2+^ and Mg^2+^, then lysed in 350 µl RLT buffer supplemented with DTT (Qiagen, Valencia, CA). Total RNA, including the microRNA (miRNA) fraction, was purified with the RNeasy Plus Mini kit (Qiagen, Valencia, CA).

Expression profiling of NF-κB pathway-associated genes was carried out using the RT^2^ Profiler™ PCR Array for Human NF-κB Signaling Pathway Plus (PAHS-025Y, Qiagen, Valencia, CA). First-strand cDNA was generated with the RT^2^ First Strand kit (Qiagen, Valencia, CA) in a GeneAmp PCR System 9700 thermal cycler (Applied Biosystems, Foster City, CA).

TLR and PCDHG transcript levels and miRNA expression were determined by quantitative reverse-transcription PCR (RT-qPCR). For MIR133A2, MIR448, and MIR518C, Applied Biosystems TaqMan Pre-miRNA assays were used. These assays employ a two-step RT-qPCR format with predesigned forward and reverse primers and a TaqMan MGB probe targeting the corresponding pre-miRNA stem-loop sequence. cDNA was synthesized with a High-Capacity cDNA Reverse Transcription kit using random hexamer primers (Applied Biosystems, Foster City, CA). Amplification was performed on a QuantStudio 12K Flex Real-Time PCR System (Applied Biosystems, Foster City, CA) using the TaqMan assays listed in the table below.

**Table:** 

Gene symbol	qPCR TaqMan assay	Manufacturer
MIR133A2	Hs04233528_s1	Thermo Fisher Scientific
MIR448	Hs04231583_s1	Thermo Fisher Scientific
MIR518C	Hs06627658_g1	Thermo Fisher Scientific
PCDHGA10	Hs00259371_s1	Thermo Fisher Scientific
PCDHGA11	Hs01107482_m1	Thermo Fisher Scientific
PCDHGA4	Hs00259326_s1	Thermo Fisher Scientific
PCDHGA5	Hs00259333_s1	Thermo Fisher Scientific
PCDHGA6	Hs00259344_s1	Thermo Fisher Scientific
PCDHGA7	Hs00259352_s1	Thermo Fisher Scientific
PCDHGA9	Hs00259365_s1	Thermo Fisher Scientific
PCDHGB1	Hs00251712_m1	Thermo Fisher Scientific
PCDHGC4	Hs00219044_m1	Thermo Fisher Scientific
PCDHGC5	Hs00260846_s1	Thermo Fisher Scientific
TLR1	Hs00413978_m1	Thermo Fisher Scientific
TLR2	Hs00152932_m1	Thermo Fisher Scientific
TLR3	Hs01551079_g1	Thermo Fisher Scientific
TLR7	Hs01933259_s1	Thermo Fisher Scientific
TLR8	Hs07292888_s1	Thermo Fisher Scientific

### Cytokine measurements

Following the 24-hour stimulation period, conditioned media were harvested and cleared of cellular debris by centrifugation at 500 × g for 5 minutes, then aliquoted and stored at −80 °C. Cell monolayers were solubilized in 200 µl RIPA buffer (Sigma-Aldrich, Saint Louis, MO) containing 1 × cOmplete EDTA-free protease inhibitor cocktail (Roche, Basel, Switzerland). Lysates were centrifuged at 12,000 rpm for 10 minutes to remove cell debris, and total protein was quantified by the bicinchoninic acid assay (ThermoFisher Scientific, Waltham, MA).

Concentrations of ten cytokines—interferon-γ (IFN-γ), interleukin (IL)-1β, IL-2, IL-4, IL-6, IL-8, IL-10, IL-12p70, IL-13, and tumor necrosis factor-α (TNF-α)—were measured simultaneously using an ultrasensitive electrochemiluminescence multiplex platform (V-PLEX Proinflammatory Panel 1 Human Kit, Meso Scale Diagnostics, Rockville, MD). Signal intensities were acquired on an MSD QuickPlex SQ120 instrument (Meso Scale Diagnostics, Rockville, MD) according to the manufacturer’s protocol, and cytokine concentrations were normalized to total cellular protein content for each sample.

### Statistics

For the RT^2^ PCR array, data analysis was performed through the Qiagen web-based PCR Array Data Analysis platform (www.SABiosciences.com/pcrarraydataanalysis.php). In brief, cycle-threshold (CT) values were normalized using an automatically selected panel of reference genes from the full array plate; the geometric mean of their CT values was used for ΔΔCT calculations. Fold regulation and nominal p-values were calculated using the QIAGEN web-based PCR Array Data Analysis platform. Statistical significance for individual genes was initially evaluated using two-tailed Student’s t-tests on replicate 2^-ΔCt values. Volcano plots were generated directly by the QIAGEN platform and therefore display fold regulation in relation to nominal p-values. To account for multiple testing across the predefined gene panel, nominal p-values were additionally adjusted using the Benjamini–Hochberg false discovery rate (FDR) procedure separately for each agonist-versus-control comparison. Genes with an FDR-adjusted p-value (q-value) < 0.05 were considered statistically significant in the gene-level analyses reported in Table 1 and the Results section.

**Table 1:** Endothelial cell regulation of NFκB pathway-associated genes by Pam3CSK4, Poly I:C or R848 compared to control.

Gene Symbol	Fold regulation Pam3CSK4	FDR q-value	Fold regulation Poly I:C	FDR q-value	Fold regulation R848	FDR q-value	
AKT1	2.4-fold decrease	0.046	n.s.		n.s.		
ATF1	11.5-fold decrease	0.002	n.s.		1.9-fold increase	0.01	
BCL2A1	n.s.		10.9-fold increase	0.04	n.s.		
BCL3	3.9-fold increase	0.005	5.5-fold increase	0.03	1.9-fold increase	0.03	
BIRC2	2.3-fold increase	0.0008	n.s.		2.5-fold increase	0.02	
BIRC3	4.4-fold increase	0.002	144-fold increase	0.03	n.s.		
CASP1	n.s.		7.4-fold increase	0.04	n.s.		
CCL2	4.7-fold increase	0.04	n.s.		n.s.		
CCL5	41-fold increase	0.003	14.4-fold decrease	0.01	n.s.		
CCL20	9.7-fold increase	0.002	866-fold increase	0.04	n.s.		
CD40	n.s.		646-fold increase	0.004	151-fold increase	0.008	
CD83	13.5-fold decrease	0.003	n.s.		n.s.		
CFLAR	2.6-fold decrease	0.0008	n.s.		n.s.		
CSF1	11-fold increase	0.002	32.3-fold increase	0.04	5.8-fold increase	0.01	
CSF2	15.2-fold increase	0.0008	533-fold increase	0.03	3.4-fold increase	0.047	
CSF3	281-fold increase	0.0001	n.s.		n.s.		
CHUK	1.7-fold increase	0.03	2.9-fold decrease	0.01	n.s.		
CXCL2	41.1-fold increase	0.02	10.9-fold increase	0.03	n.s.		
CXCL3	17.3-fold increase	0.001	18.2-fold increase	0.04	n.s.		
CXCL8	4.6-fold increase	0.01	15.7-fold increase	0.04	n.s.		
EGFR	n.s.		6.9-fold increase	0.04	2.2-fold increase	0.01	
EGR1	n.s.		2.7-fold decrease	0.01	n.s.		
ELK1	4.1-fold increase	0.02	1.9-fold increase	0.03	7.5-fold increase	0.047	
FOS	2.3-fold decrease	0.04	4-fold increase	0.04	n.s.		
HMOX1	2-fold decrease	0.04	n.s.		n.s.		
ICAM1	9.5-fold increase	0.02	21.7-fold increase	0.02	n.s.		
IFNA1	n.s.		2.5-fold decrease	0.03	n.s.		
IKBKB	3.4-fold decrease	0.04	2.9-fold decrease	0.04	n.s.		
IKBKG	2.2-fold decrease	0.01	7.3-fold decrease	0.006	n.s.		
IL1R1	4.9-fold increase	0.0001	n.s.		2.3-fold increase	0.02	
IRAK2	1.6-fold increase	0.046	9.3-fold increase	0.03	3.2-fold decrease	0.01	
IRF1	n.s.		6.2-fold increase	0.03	3.1-fold decrease	0.04	
JUN	n.s.		3.6-fold decrease	0.02	3.3-fold decrease	0.02	
LTBR	1.6-fold increase	0.04	n.s.		n.s.		
MALT1	10.4-fold decrease	0.02	3.8-fold decrease	0.04	n.s.		
MAP3K1	5.8-fold decrease	0.009	5.8-fold decrease	0.02	n.s.		
MYD88	18.2-fold increase	0.008	31.7-fold increase	0.02	5.7-fold increase	0.047	
NFKBIA	3.3-fold increase	0.0008	8.2-fold increase	0.03	n.s.		
NFKBIE	3.6-fold increase	0.02	6.3-fold increase	0.007	n.s.		
NOD1	9.8-fold decrease	0.04	3.8-fold decrease	0.03	2.8-fold decrease	0.04	
PSIP1	6.8-fold decrease	0.0008	3.5-fold decrease	0.01	n.s.		
RELB	14.1-fold increase	0.0008	20.8-fold increase	0.03	n.s.		
STAT1	1.6-fold decrease	0.04	n.s.		1.8-fold decrease	0.047	
STX11	n.s.		3.3-fold increase	0.03	2-fold decrease	0.02	
TBK1	2.3-fold increase	0.008	n.s.		n.s.		
TICAM1	n.s.		2-fold decrease	0.03	2.6-fold decrease	0.006	
TLR1	n.s.		n.s.		7.7-fold decrease	0.04	
TLR2	12-fold increase	0.02	32.5-fold increase	0.03	n.s.		
TLR3	2.2-fold increase	0.02	13-fold increase	0.03	1.6-fold increase	0.04	
TLR4	n.s.		6-fold decrease	0.04	n.s.		
TLR6	n.s.		4.6-fold decrease	0.02	n.s.		
TNFAIP2	2-fold increase	0.03	n.s.		n.s.		
TNFAIP3	1.8-fold increase	0.02	11.3-fold increase	0.03	1.9-fold decrease	0.02	
TNFRSF10B	2.5-fold decrease	0.02	4-fold decrease	0.01	n.s.		
TNFSF10	5.5-fold increase	0.01	43-fold increase	0.04	n.s.		
TRADD	n.s.		3.7-fold increase	0.005	n.s.		
TRAF2	2-fold increase	0.007	n.s.		2-fold increase	0.04	
TRAF6	2.3-fold increase	0.03	n.s.		n.s.		

n = 3–4.

n.s = not significantly different compared to controls.

Blue indicates genes only altered by Pam3CSK4, Yellow indicates genes only altered by Poly I:C, Red indicates genes only altered by R848, Green indicates genes upregulated by all agonists, Pink indicates genes regulated by both Pam3CSK4 and Poly I:C.

Global expression patterns were explored by principal component analysis (PCA) using Qlucore Omics Explorer 3.9 (Qlucore AB, Lund, Sweden).

TLR, PCDHG, and miRNA transcript levels were normalized to POLR2A. Relative expression following stimulation was calculated using the 2^-ΔΔCt method, with untreated cells used as the calibrator. Reactions with no detectable amplification were recorded as undetermined and were assigned an arbitrary Ct value of 40. Targets that were undetectable in all biological replicates of a condition were considered not detected. qPCR reactions were performed in technical duplicate, and the technical replicates were averaged before statistical analysis. TLR-, PCDHG- and miR expression data and cytokine secretion levels are presented as means ± standard deviation (SD). Group comparisons were performed by one-way analysis of variance (ANOVA) followed by Tukey’s multiple-comparisons test using GraphPad Prism version 10 (GraphPad Software, San Diego, CA), with statistical significance set at p < 0.05.

## Results

### Expression of TLR1, TLR2, TLR3, TLR7, and TLR8 in human cardiac endothelial cells

To characterize expression of the receptors targeted by the agonists used in this study, TLR1, TLR2, TLR3, TLR7, and TLR8 transcript levels were determined by RT-qPCR in primary human cardiac ECs exposed for 24 hours to Pam3CSK4, Poly I:C, or R848, using LPS as a reference stimulus. TLR1, TLR2, and TLR3 were readily detected at similar levels, whereas TLR7 and TLR8 transcripts were below the limit of detection (Fig 1A, S1 and S2 Tables). Following 24 h stimulation, Poly I:C markedly increased expression of TLR2, TLR3, and TLR7, while Pam3CSK4 and R848 did not significantly alter expression of any of the TLRs examined (Fig 1B). TLR8 remained undetectable under all conditions tested. The reference stimulant LPS did not significantly alter the expression of any of the TLRs examined.

**Fig 1:**
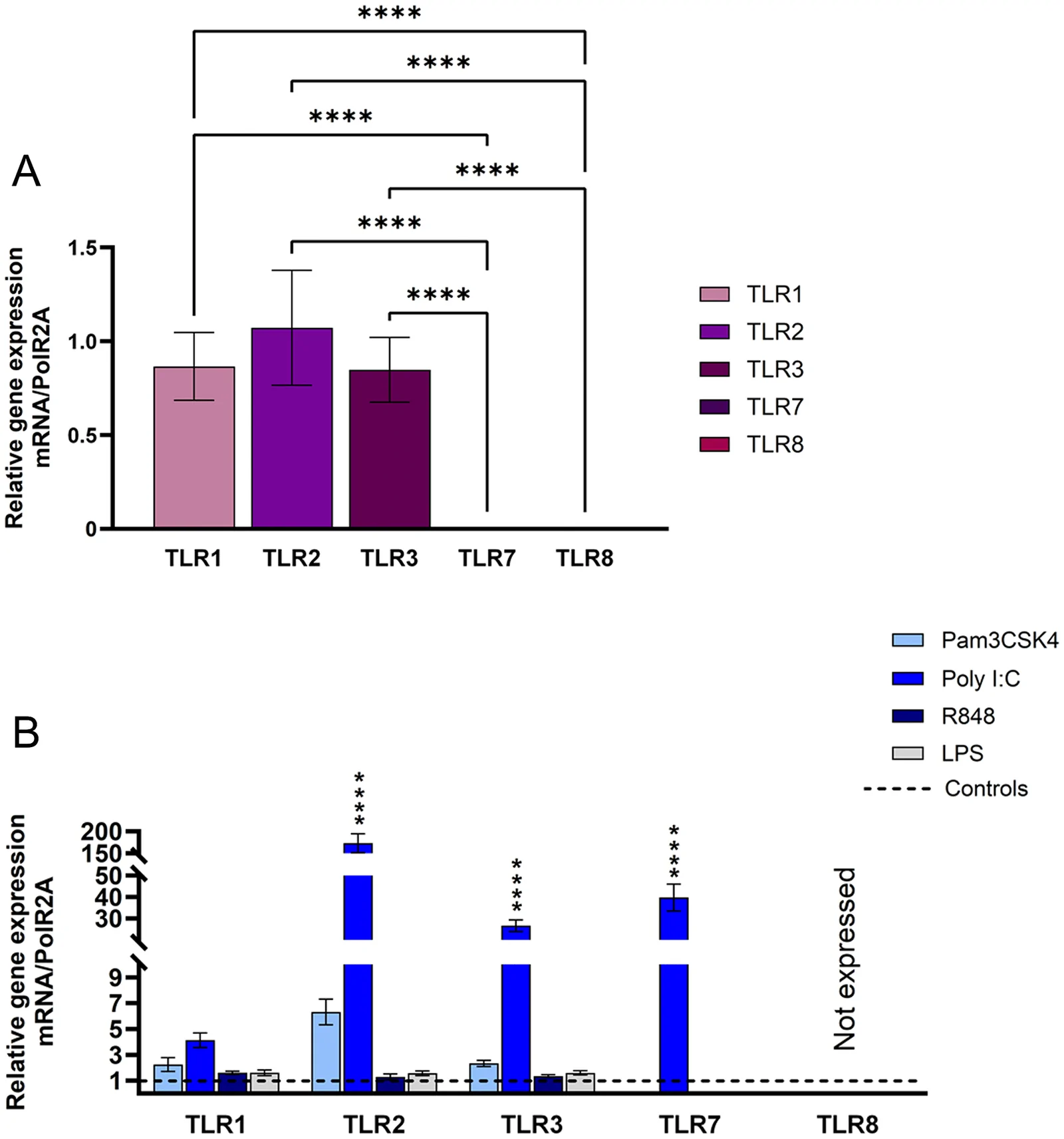
TLR gene levels in human cardiac endothelial cells following Pam3CSK4, Poly I:C, R848, or LPS stimulation. (A) Bar graph displaying relative expression levels of TLR1, TLR2, TLR3, TLR7 and TLR8 in untreated cardiac ECs, (B) Bar graph displaying relative expression levels of TLR1, TLR2, TLR3, TLR7 and TLR8 in ECs treated with Pam3CSK4, Poly I:C, R848, or LPS (reference stimulant) compared to untreated controls (dashed line). Bars show data as the mean and standard deviation (SD). n = 8, *p-value <0.05 (one-way ANOVA).

### Pam3CSK4, Poly I:C, and R848 induce distinct transcriptional profiles among NF-κB pathway-associated genes in cardiac endothelial cells

Having established the basal expression and stimulus-dependent regulation of the relevant TLRs, we next characterized the broader transcriptional responses to Pam3CSK4, Poly I:C, and R848 using a focused panel of 84 NF-κB pathway-associated genes. To this end, primary human cardiac ECs were exposed for 24 hours to Pam3CSK4, Poly I:C, or R848, each at concentrations optimized for maximal receptor engagement. Of these, six transcripts—AGT, CARD11, FASLG, IFNG, IL10, and TLR9—fell below the detection limit across all conditions and were excluded from further analysis (S3 Table).

All three agonists altered gene expression within the predefined panel relative to vehicle-treated controls; however, PCA of the complete dataset revealed clearly segregated treatment clusters, indicating that Pam3CSK4, Poly I:C, and R848 elicited distinct transcriptional profiles within the predefined gene panel (Fig 2A). Detailed gene-level inspection confirmed this divergence (Fig 2B, Table 1 and S4 Table). A core set of six genes was significantly upregulated regardless of the agonist used: BCL3 (fold regulation: Pam3CSK4, 4; Poly I:C, 5; R848, 2), CSF1 (11; 32; 6), CSF2 (15; 530; 3), ELK1 (4; 2; 8), MYD88 (18; 32; 6), and TLR3 (2; 13; 2). Conversely, NOD1 was consistently suppressed (10; 4; 3-fold decrease). IRAK2 and TNFAIP3 showed opposing regulation, with increased expression following Pam3CSK4 and Poly I:C treatment and decreased expression after R848 stimulation (Fig 2B, Table 1 and S4 Table).

**Fig 2:**
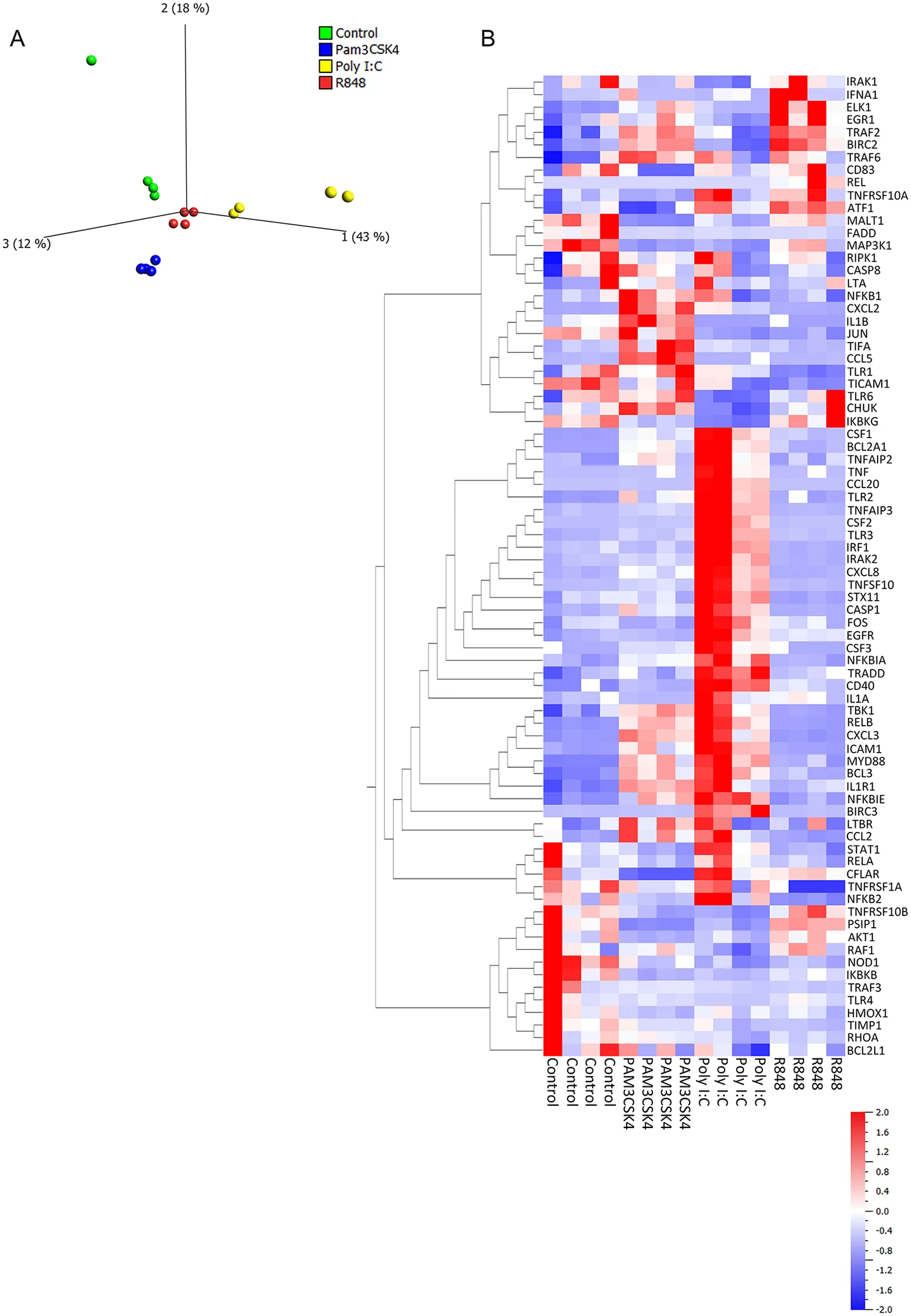
Transcriptional responses of NF-κB pathway-associated genes following Pam3CSK4, Poly I:C, or R848 stimulation of human cardiac endothelial cells. (A) PCA score plot (first three components) based on expression of the predefined panel of NF-κB pathway-associated genes, showing a separation between cardiac ECs treated with Pam3CSK4 (blue), Poly I:C (yellow) or R848 (red) and untreated control cells (green), (B) Heatmap showing expression patterns within the focused gene panel after stimulation with Pam3CSK4, Poly I:C, or R848 compared to untreated cells (controls). n = 4, log2 fold change.

### Transcriptional response to Pam3CSK4

Pam3CSK4 stimulation yielded significant upregulation of 30 transcripts and downregulation of 14 transcripts relative to untreated controls (Figs 2B and 3A, Table 1 and S4 Table). Among the most strongly induced genes were CSF3 (280-fold increase), CCL5 (41-fold increase), CXCL2 (41-fold increase), and MYD88 (18-fold increase). The most marked downregulation was observed for CD83 (14-fold decrease), ATF1 (11-fold decrease), MALT1 (10-fold decrease), and NOD1 (10-fold decrease). Ten of the 44 differentially expressed genes were uniquely altered by Pam3CSK4 and showed no significant change with Poly I:C or R848: AKT1, CCL2, CD83, CFLAR, CSF3, HMOX1, LTBR, TBK1, TNFAIP2, and TRAF6 (Table 1).

**Fig 3:**
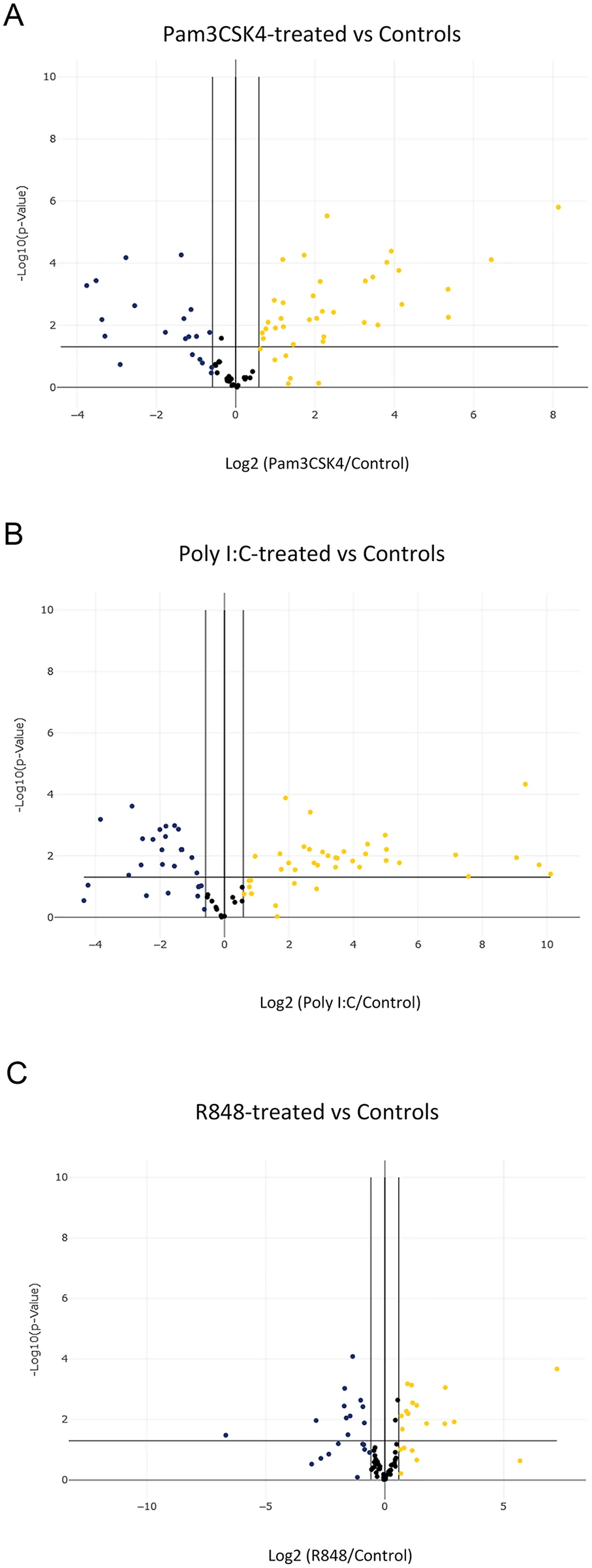
Volcano plot analysis of NF-κB pathway-associated gene expression following Pam3CSK4, Poly I:C, or R848 stimulation of human cardiac endothelial cells. (A-C) Volcano plots generated using the QIAGEN PCR Array Data Analysis platform, displaying fold regulation and nominal p-values from two-tailed Student’s t-tests for Pam3CSK4 (A), Poly I:C (B) or R848 (C) compared to untreated cells. n = 4, log2 fold change, p-value <0.05 (t-test).

### Transcriptional response to Poly I:C

Poly I:C stimulation, which also markedly increased TLR3 expression, induced extensive transcriptional changes, with 27 genes significantly elevated and 15 genes downregulated compared to controls (Figs 2B and 3B; Table 1 and S4 Table). Several transcripts showed exceptionally large magnitude changes: CCL20 (860-fold increase), CD40 (640-fold increase), CSF2 (530-fold increase), and BIRC3 (144-fold increase), while CCL5 showed a 14-fold decrease. Seven of the 42 genes with altered expression were modulated exclusively by Poly I:C and remained unchanged in response to Pam3CSK4 or R848 (Table 1); notable among these were BCL2A1, CASP1, EGR1, IFNA1, TLR4, TLR6, and TRADD (Table 1).

### Transcriptional response to R848

R848 stimulation produced the most restrained transcriptional response: 12 genes were upregulated and 9 genes were downregulated compared to controls (Figs 2B and 3C; Table 1 and S4 Table). With the exception of CD40, which exhibited a striking 150-fold increase, upregulated transcripts showed only modest changes of 1.7- to 7.5-fold. Downregulated genes of note included TLR1 (7-fold decrease), JUN (3-fold decrease), IRAK2 (3-fold decrease), and IRF (3-fold decrease). Of the 21 differentially expressed genes, only TLR1 was uniquely altered by R848 (Table 1).

### Pam3CSK4 and Poly I:C, but not R848, increase cytokine secretion from cardiac endothelial cells

Having established the transcriptomic responses, we next asked whether the observed gene-level changes translated into corresponding changes in protein secretion. Ten proinflammatory cytokines were quantified in conditioned media from cardiac ECs treated with Pam3CSK4, Poly I:C, or R848; the TLR4 agonist LPS was included as a positive reference. Across all cytokines measured, Poly I:C evoked the strongest secretory response, with fold increases ranging from 22-fold to 670-fold over baseline (Fig 4 and S5 Table). Pam3CSK4 also robustly stimulated cytokine release, producing 10- to 68-fold elevations (Fig 4). In contrast, R848 failed to significantly alter the concentration of any cytokine relative to control conditions (Fig 4). The reference compound LPS had comparatively modest effects in cardiac ECs; nonetheless, it significantly elevated IL-1β, IL-2, and IL-13 above control levels (Fig 4A–B, H). IFN-γ was undetectable in R848-treated cultures and was present only at trace levels even after Poly I:C exposure (Fig 4J). Following Poly I:C stimulation, IL-1β, IL-2, IL-4, IL-10, and IL-12p70 accumulated to concentrations in the range of 100–400 pg/mg protein (Fig 4A–C, F–G), whereas IL-6, IL-8, IL-13, and TNF-α reached markedly higher concentrations, from approximately 2,500 pg/mg protein for TNF-α to roughly 1 µg/mg protein for IL-8 (Fig 4D–E, H–I).

**Fig 4:**
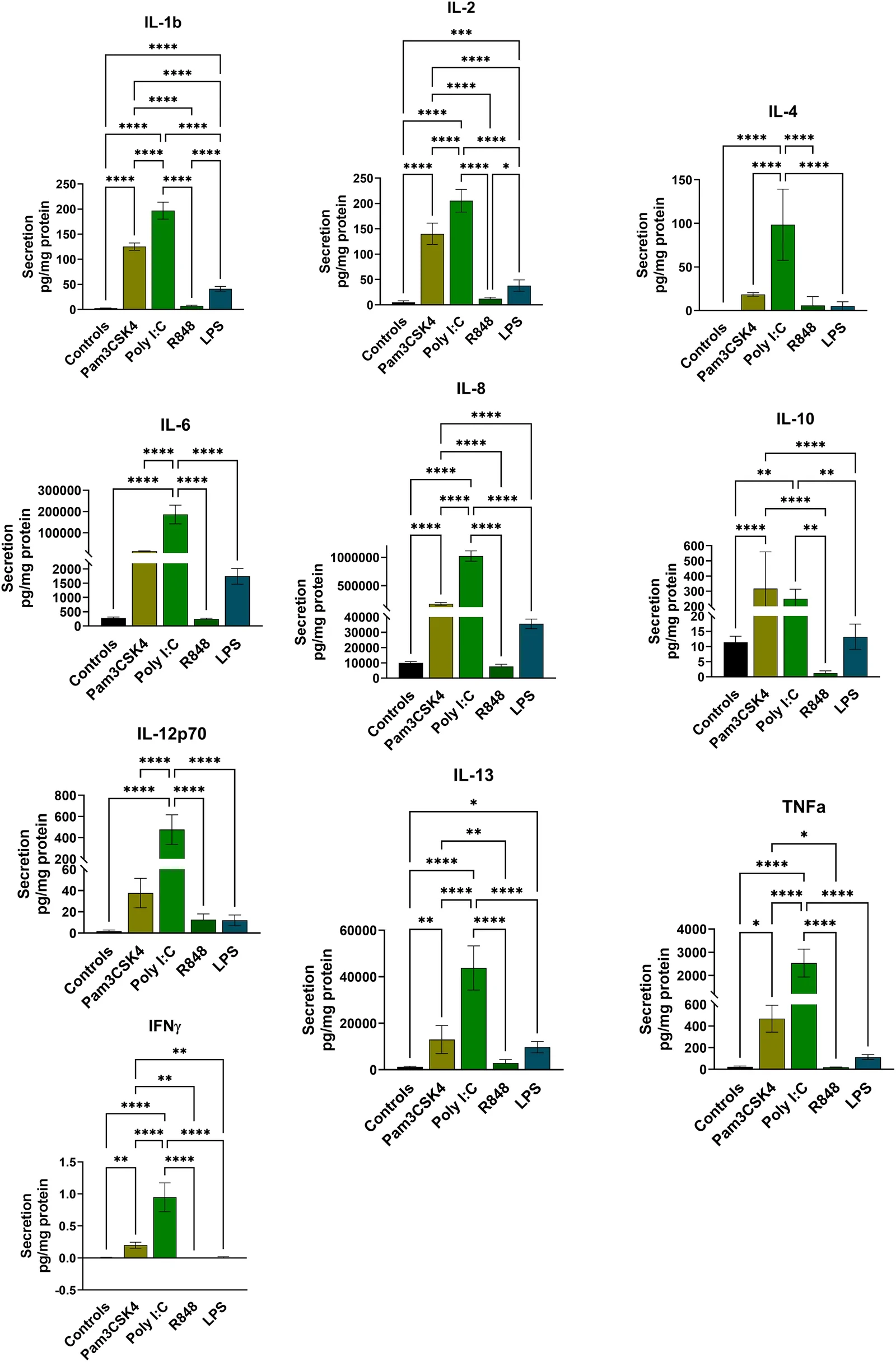
Concentration of proinflammatory cytokines secreted from human cardiac endothelial cells following Pam3CSK4, Poly I:C, or R848 stimulation. Cytokine levels were measured in conditioned medium following treatment of cardiac ECs with Pam3CSK4, Poly I:C or R848 and compared to medium from untreated cells (controls). Bars show data as the mean and standard deviation (SD). n = 7-8. *p < 0.05, **p < 0.01, ***p < 0.001, ****p < 0.0001 (one way ANOVA).

Collectively, these data demonstrate that Pam3CSK4 and Poly I:C induce pronounced cytokine secretion from cardiac ECs, whereas R848 does not significantly alter cytokine secretion under the conditions tested.

### Poly I:C decreases γ-protocadherin transcript levels in cardiac endothelial cells

Our earlier work in myocardial biopsies from patients with severe coronary artery disease or valvular heart disease revealed concurrent downregulation of miR-133A/B, miR-448, and miR-518C alongside reduced γ-protocadherin (PCDHG) transcript levels [17]. The same tissue samples exhibited elevated expression of TLR1, TLR3, and TLR7 relative to non-diseased controls [16]. We therefore explored whether direct activation of these TLRs could modulate miRNA and PCDHG expression in cardiac ECs, using LPS as a reference stimulus.

None of the three miRNAs—miR-133A2, miR-448, or miR-518C—were detectable in the endothelial cultures (S6 Table). Likewise, PCDHGC4 and PCDHGC5 transcripts were below the limit of detection at baseline (S6 Table). Among the remaining PCDHG family members, Poly I:C stimulation triggered a pronounced and uniform downregulation, reducing transcript abundance by 46–100% (Fig 5 and S7 Table). By contrast, Pam3CSK4 unexpectedly increased expression of all but two PCDHG genes, an effect mirrored by the reference agonist LPS (Fig 4 and S7 Table). R848 had no appreciable effect on PCDHG expression (Fig 5 and S7 Table).

**Fig 5:**
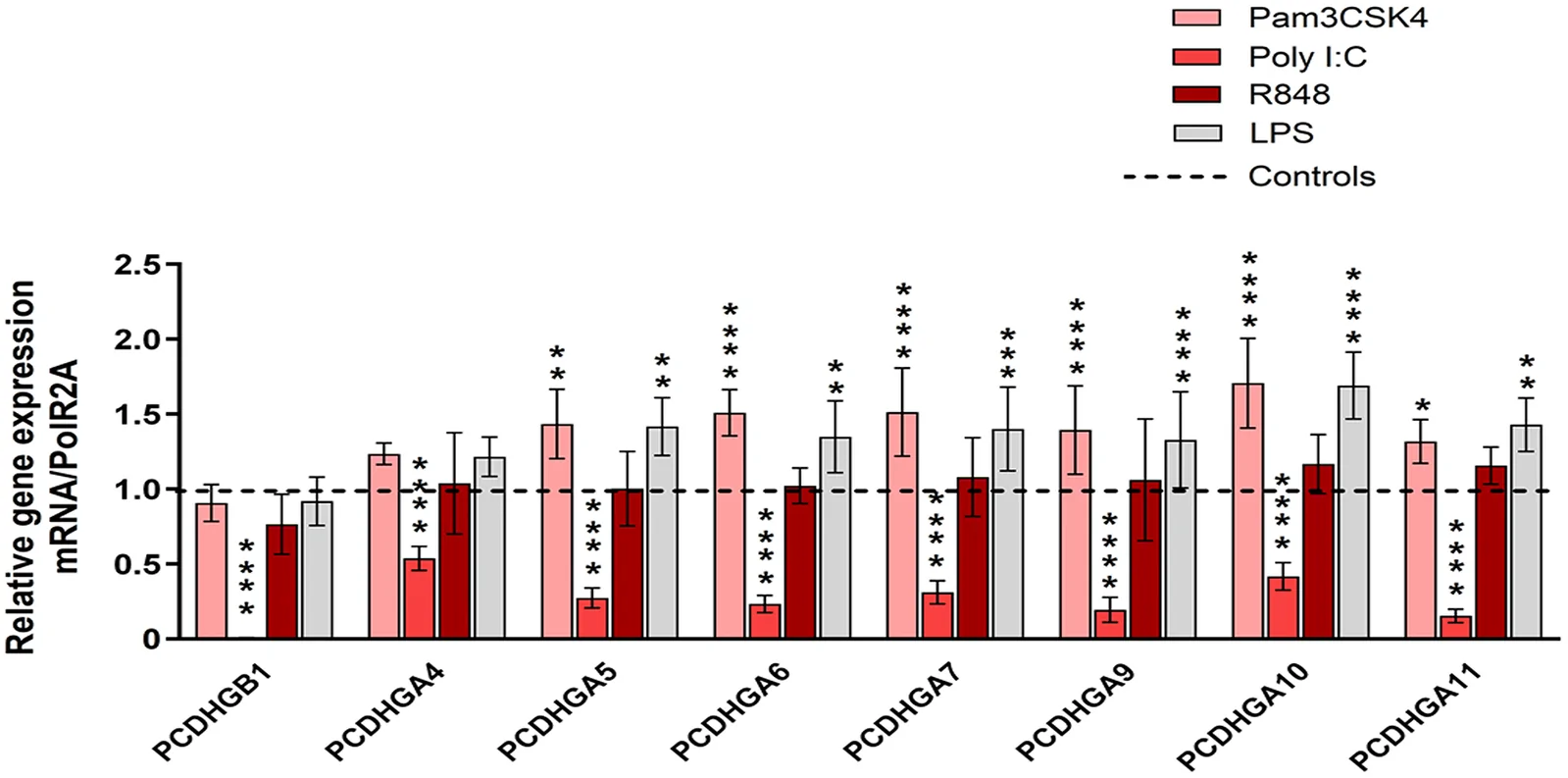
γ-protocadherin gene levels in human cardiac endothelial cells following Pam3CSK4, Poly I:C, R848, or LPS stimulation. Bar graph displaying relative expression levels of γ-protocadherin genes. The dashed line indicates gene expression levels of untreated cardiac ECs (controls), bars represent gene expression levels of ECs treated with Pam3CSK4, Poly I:C, R848 or LPS (reference stimulant). Bars show data as the mean and standard deviation (SD). n = 8, *p-value <0.05 (one way ANOVA).

Taken together, Poly I:C markedly downregulated PCDHG expression in cardiac ECs, whereas Pam3CSK4 generally increased these transcripts and R848 had no significant effect. MIR133A2, MIR448, and MIR518C transcripts were not detected using the TaqMan Gene Expression assays employed in this study. The PCDHG responses therefore occurred without detectable expression of these MIR transcripts under the experimental conditions examined.

## Discussion

The experiments described here extend our earlier tissue-level observations in patients with advanced coronary artery disease and aortic valve disease, in whom TLR1, TLR3, and TLR7, together with their downstream effectors, were upregulated while PCDHG levels were reduced compared to healthy myocardium [16,17]. By stimulating primary human cardiac ECs with Pam3CSK4, Poly I:C, and R848, we show that these TLR agonists induce distinct transcriptional profiles among NF-κB pathway-associated genes and differentially affect cytokine secretion and PCDHG expression, suggesting that TLR1/2, TLR3, and TLR7/8 pathways elicit distinct molecular responses in the human heart.

ECs are increasingly recognized as important innate immune sentinels capable of detecting pathogen-associated and danger-associated molecular patterns through a repertoire of pattern-recognition receptors, including TLRs [8,21–23]. Activation of these receptors promotes expression of cytokines, chemokines, and adhesion molecules that orchestrate leukocyte recruitment and vascular inflammation [24–27]. Dysregulated or sustained endothelial TLR activity is implicated in the progression of cardiovascular diseases, including atherosclerosis, heart failure, and coronary microvascular dysfunction [2–4,28–30].

TLR3 is expressed in various types of ECs and recognizes double-stranded RNA, such as viral RNA or synthetic analogs like Poly I:C. In the current study, TLR3 was readily detectable in untreated cardiac ECs at levels comparable to TLR1 and TLR2, and its expression increased markedly following Poly I:C stimulation. Poly I:C also induced an extensive transcriptional response and the strongest secretory response among the three stimuli tested. IL-6, IL-8, TNFα, and IL-1β secretion was markedly increased, accompanied by pronounced transcriptional changes in genes including CSF2 and CD40. Together, the constitutive expression of TLR3, its strong induction by Poly I:C, and the magnitude of the downstream inflammatory response are consistent with a prominent role for TLR3-associated signaling in cardiac ECs. However, Poly I:C also increased TLR2 and TLR7 expression, indicating that double-stranded RNA stimulation alters the broader TLR repertoire of these cells. Previous studies have similarly shown that Poly I:C can impair endothelium-dependent vasodilation and promote vascular inflammation [23,31], and TLR3 has been implicated more broadly in the regulation of vascular and endothelial function [24,32]. In addition, although Poly I:C is widely used as a TLR3 agonist, it can also activate cytosolic RNA-sensing pathways, including RIG-I-like receptors such as MDA5 [33]. The responses observed here therefore cannot be attributed exclusively to TLR3 signaling.

TLR1 forms heterodimers with TLR2 to recognize triacylated lipopeptides, whereas TLR2/TLR6 heterodimers preferentially recognize diacylated lipopeptides. Genetic polymorphisms in TLR1 have been linked to altered inflammatory signaling and various immune-related diseases [34]. Pam3CSK4 is a synthetic triacylated lipopeptide that signals through TLR1/TLR2 heterodimers. In the present study, both TLR1 and TLR2 were readily detectable in untreated cardiac ECs at comparable levels, and their expression was not significantly altered by Pam3CSK4 stimulation. Nevertheless, Pam3CSK4 induced a pronounced inflammatory response, including increased expression of MYD88, CCL5, and CXCL2 and significant secretion of IL-1β, IL-2, IL-6, IL-8, and TNFα. Several genes, including AKT1, LTBR, and TRAF6, were uniquely regulated by Pam3CSK4, demonstrating a transcriptional profile that was partially distinct from those induced by Poly I:C and R848. These findings are consistent with previous studies of TLR1/TLR2-directed stimulation in endothelial cells. Liu et al. reported that a TLR1/TLR2 agonist induced a distinct transcriptional profile related to cytokine production and regulation in liver sinusoidal endothelial cells, differing from responses to Poly I:C, LPS, or IFN-γ and promoting T-cell proliferation and activation [35].

R848 stimulation elicited a comparatively limited response in cardiac ECs, with relatively few changes among NF-κB pathway-associated genes and no significant increase in secretion of the cytokines examined. Although CD40 expression was markedly upregulated, most other transcripts were only modestly affected, and IRAK2 and TNFAIP3 were downregulated. The absence of detectable TLR7 and TLR8 transcripts at baseline may contribute to the comparatively limited R848 response in cardiac ECs, although transcript abundance alone does not establish functional receptor availability; moreover, TLR7/8-independent effects of R848 cannot be excluded. The low basal expression of TLR7 is consistent with studies suggesting that endothelial TLR7 expression is low or condition-dependent [36–38]. TLR7 has been detected in microvascular ECs within human atherosclerotic plaques [37], whereas it is absent or expressed at very low levels in other endothelial populations [36]. Interestingly, Poly I:C markedly induced TLR7 expression from undetectable baseline levels, whereas TLR8 remained undetectable, indicating that TLR7 expression in cardiac ECs can be induced by inflammatory stimulation. In our previous study, TLR7 expression was significantly elevated in myocardial tissue from both ischemic and non-ischemic heart disease patients [16]. The present findings therefore raise the possibility that endothelial TLR7 expression is dynamically regulated by the inflammatory environment, although the mechanisms responsible for its induction in diseased myocardium remain to be established.

Together, these findings highlight the cell-type- and stimulus-dependent nature of innate immune responses in cardiac ECs. The distinct transcriptional and secretory profiles induced by Pam3CSK4, Poly I:C, and R848 occurred in the context of constitutive TLR1, TLR2, and TLR3 expression, whereas TLR7 was inducible and TLR8 remained undetectable. Thus, both receptor availability and stimulus-dependent changes in the TLR repertoire may influence how cardiac ECs respond to different innate immune stimuli, with potential implications for vascular inflammation and cardiac pathology.

In the present study, Pam3CSK4, Poly I:C, and R848 produced markedly different effects on PCDHG expression in cardiac ECs. Poly I:C caused a pronounced downregulation of all detectable PCDHG transcripts, whereas Pam3CSK4 increased expression of all but two PCDHG genes. R848 did not significantly alter PCDHG expression. These contrasting responses indicate that PCDHG regulation in cardiac ECs is strongly stimulus-dependent. Notably, the miRNAs previously associated with reduced PCDHG expression in diseased myocardium—miR-133A2, miR-448, and miR-518C—were undetectable in cardiac ECs using the TaqMan Gene Expression assays employed here. The Poly I:C-associated reduction in PCDHG expression therefore occurred without detectable expression of these MIR transcripts under the experimental conditions examined.

The functional role of PCDHGs in endothelial cells remains incompletely understood. Studies in different vascular beds suggest that individual γ-protocadherins can exert distinct, and in some cases opposing, effects on endothelial inflammatory signaling and vascular function. For example, altered PcdhgC3 expression has been associated with changes in inflammatory responsiveness in brain microvascular ECs, whereas endothelial PCDHGA9 has been implicated in pro-atherogenic signaling in aortic ECs [14,15]. These observations indicate that the consequences of altered PCDHG expression are likely to depend on the specific family member, endothelial subtype, and inflammatory or vascular context.

Our findings add another level of complexity by showing that the direction of PCDHG regulation in human cardiac microvascular ECs differs markedly depending on the inflammatory stimulus. The strong reduction induced by Poly I:C is consistent with our previous observations of reduced PCDHG expression in diseased myocardium and following Poly I:C stimulation of cardiac fibroblasts [17,18]. In contrast, the increase in PCDHG expression following Pam3CSK4 stimulation and the absence of an effect of R848 demonstrate that reduced PCDHG expression is not a general consequence of innate immune stimulation. Importantly, the present experiments do not establish whether these transcriptional changes are protective, maladaptive, or directly involved in endothelial dysfunction. Functional studies will therefore be required to determine whether stimulus-dependent changes in PCDHG expression influence processes such as endothelial barrier integrity, migration, angiogenesis, and inflammatory activation in the cardiac microvasculature.

Comparison with our previous cardiac fibroblast study [18] reveals both shared and cell-type-specific responses. Poly I:C produced a strong transcriptional response and decreased PCDHG expression in both cardiac ECs and fibroblasts, suggesting that sensitivity to double-stranded RNA-directed stimulation is a shared feature of these two cardiac stromal cell types. However, several important differences were observed. Pam3CSK4 generally increased PCDHG expression in ECs, whereas it decreased selected PCDHG transcripts in fibroblasts. R848 did not significantly alter cytokine secretion or PCDHG expression in ECs, whereas it produced a broader transcriptional response, selectively increased several cytokines, and increased *PCDHGA5* and *PCDHGA9* expression in fibroblasts. The overall response patterns also differed between the two cell types, particularly for Pam3CSK4 and R848.

These cell-type differences are biologically relevant because fibroblasts and ECs have distinct functions within the myocardium. Fibroblast responses are closely linked to extracellular-matrix remodeling, scar formation, and fibrosis, whereas EC responses can directly influence microvascular permeability, vascular tone, leukocyte recruitment, tissue perfusion, and paracrine communication with cardiomyocytes and other cardiac cells. The current study therefore extends the previous fibroblast findings by identifying how the same innate immune stimuli are interpreted by the cardiac microvascular endothelium. In particular, the limited R848 response and the opposing effects of Pam3CSK4 on PCDHG expression represent endothelial-specific findings that were not predictable from the fibroblast study.

Some limitations should be considered. First, only a 24-hour stimulation period was examined, and the comparatively limited response to R848 may not reflect transient effects occurring at earlier time points. Second, the focused PCR array measures a predefined set of pathway-associated transcripts and therefore does not provide a genome-wide transcriptional profile or directly demonstrate NF-κB activation or dependence. Third, although Pam3CSK4 and R848 are established agonists of TLR1/TLR2 and TLR7/TLR8, respectively, the individual contribution of each receptor cannot be determined from the present experiments. Both TLR1 and TLR2 were readily detectable in untreated cardiac ECs, supporting the presence of the receptor pair targeted by Pam3CSK4. In contrast, TLR7 and TLR8 transcripts were undetectable at baseline, and neither was induced by R848, although Poly I:C markedly induced TLR7 expression. These findings indicate that receptor expression in cardiac ECs is stimulus-dependent, but transcript abundance alone does not establish functional receptor availability or receptor-specific causality.

In summary, Pam3CSK4, Poly I:C, and R848 induced distinct transcriptional, cytokine, and PCDHG responses in primary human cardiac microvascular ECs. Poly I:C produced the strongest inflammatory response and the most pronounced reduction in PCDHG expression under the conditions tested, whereas R848 produced comparatively limited effects at 24 hours. The receptor-expression data further show that cardiac ECs constitutively express TLR1, TLR2, and TLR3, whereas TLR7 expression is inducible and TLR8 remained undetectable. Comparison with cardiac fibroblasts demonstrates that responses to these innate immune stimuli are strongly cell-type-dependent, particularly with respect to R848 responsiveness and PCDHG regulation. Together, these findings support a prominent role for RNA-sensing pathways in cardiac endothelial inflammation and provide a foundation for investigating how microbial and endogenous RNA-associated stimuli influence inflammatory responses in the cardiac microvasculature.

## Supporting information

S1 Table CT values for the TLR genes for all samples included in Fig 1. (XLSX)

S2 Table Relative quantative values of TLR genes for all samples presented in Fig 1. (XLSX)

S3 Table CT values for the Human NF-κB Signaling Pathway Plus RT^2^ Profiler™ PCR Array. (XLSX)

S4 Table Relative quantative values of the genes included in the Human NF-κB Signaling Pathway Plus RT^2^ Profiler™ PCR Array. (XLSX)

S5 Table Cytokine concentrations values for all samples included in Fig 3. (XLSX)

S6 Table CT values for the PCDHG genes and miR genes investigated. (XLSX)

S7 Table Relative quantative values of PCDHG genes for all samples presented in Fig 4. (XLSX)

## References

[1] Litviňuková M, Talavera-López C, Maatz H, Reichart D, Worth CL, Lindberg EL, et al. Cells of the adult human heart. Nature. 2020;588(7838):466–72. doi: 10.1038/s41586-020-2797-4 32971526 PMC7681775

[2] Cornuault L, Rouault P, Duplàa C, Couffinhal T, Renault M-A. Endothelial dysfunction in heart failure with preserved ejection fraction: What are the experimental proofs? Front Physiol. 2022;13:906272. doi: 10.3389/fphys.2022.906272 35874523 PMC9304560

[3] Medina-Leyte DJ, Zepeda-Garcia O, Dominguez-Perez M, Gonzalez-Garrido A, Villarreal-Molina T, Jacobo-Albavera L. Endothelial dysfunction, inflammation and coronary artery disease: Potential biomarkers and promising therapeutical approaches. Int J Mol Sci. 2021;22(8). 10.3390/ijms22083850 PMC8068178 33917744

[4] Alexander Y, Osto E, Schmidt-Trucksäss A, Shechter M, Trifunovic D, Duncker DJ, et al. Endothelial function in cardiovascular medicine: A consensus paper of the European Society of Cardiology Working Groups on Atherosclerosis and Vascular Biology, Aorta and Peripheral Vascular Diseases, Coronary Pathophysiology and Microcirculation, and Thrombosis. Cardiovasc Res. 2021;117(1):29–42. doi: 10.1093/cvr/cvaa085 32282914 PMC7797212

[5] Hsieh PCH, Davis ME, Lisowski LK, Lee RT. Endothelial-cardiomyocyte interactions in cardiac development and repair. Annu Rev Physiol. 2006;68:51–66. doi: 10.1146/annurev.physiol.68.040104.124629 16460266 PMC2754585

[6] Kivelä R, Hemanthakumar KA, Vaparanta K, Robciuc M, Izumiya Y, Kidoya H, et al. Endothelial cells regulate physiological cardiomyocyte growth via VEGFR2-mediated paracrine signaling. Circulation. 2019;139(22):2570–84. doi: 10.1161/CIRCULATIONAHA.118.036099 30922063 PMC6553980

[7] Nicin L, Wagner JUG, Luxán G, Dimmeler S. Fibroblast-mediated intercellular crosstalk in the healthy and diseased heart. FEBS Lett. 2022;596(5):638–54. doi: 10.1002/1873-3468.14234 34787896

[8] Khakpour S, Wilhelmsen K, Hellman J. Vascular endothelial cell Toll-like receptor pathways in sepsis. Innate Immun. 2015;21(8):827–46. doi: 10.1177/1753425915606525 26403174

[9] Sano K, Tanihara H, Heimark RL, Obata S, Davidson M, St John T, et al. Protocadherins: A large family of cadherin-related molecules in central nervous system. EMBO J. 1993;12(6):2249–56. doi: 10.1002/j.1460-2075.1993.tb05878.x 8508762 PMC413453

[10] Nollet F, Kools P, van Roy F. Phylogenetic analysis of the cadherin superfamily allows identification of six major subfamilies besides several solitary members. J Mol Biol. 2000;299(3):551–72. doi: 10.1006/jmbi.2000.3777 10835267

[11] Onouchi T, Kishino-Kaneko Y, Kameshita I, Ishida A, Sueyoshi N. Regulation of Ca(2+)/calmodulin-dependent protein kinase phosphatase (CaMKP/PPM1F) by protocadherin-γC5 (Pcdh-γC5). Arch Biochem Biophys. 2015;585:109–20. doi: 10.1016/j.abb.2015.09.014 26386307

[12] Pancho A, Aerts T, Mitsogiannis MD, Seuntjens E. Protocadherins at the crossroad of signaling pathways. Front Mol Neurosci. 2020;13:117. doi: 10.3389/fnmol.2020.00117 32694982 PMC7339444

[13] Dilling C, Roewer N, Förster CY, Burek M. Multiple protocadherins are expressed in brain microvascular endothelial cells and might play a role in tight junction protein regulation. J Cereb Blood Flow Metab. 2017;37(10):3391–400. doi: 10.1177/0271678X16688706 28094605 PMC5624389

[14] Gabbert L, Dilling C, Meybohm P, Burek M. Deletion of protocadherin gamma C3 induces phenotypic and functional changes in brain microvascular endothelial cells in vitro. Front Pharmacol. 2020;11:590144. doi: 10.3389/fphar.2020.590144 33390965 PMC7774295

[15] Joshi D, Coon BG, Chakraborty R, Deng H, Yang Z, Babar MU, et al. Endothelial γ-protocadherins inhibit KLF2 and KLF4 to promote atherosclerosis. Nat Cardiovasc Res. 2024;3(9):1035–48. doi: 10.1038/s44161-024-00522-z 39232138 PMC11399086

[16] Rotter Sopasakis V, Sandstedt J, Johansson M, Lundqvist A, Bergström G, Jeppsson A, et al. Toll-like receptor-mediated inflammation markers are strongly induced in heart tissue in patients with cardiac disease under both ischemic and non-ischemic conditions. Int J Cardiol. 2019;293:238–47. doi: 10.1016/j.ijcard.2019.06.033 31230935

[17] Sandstedt J, Vukusic K, Rekabdar E, Dellgren G, Jeppsson A, Mattsson Hultén L, et al. Markedly reduced myocardial expression of γ-protocadherins and long non-coding RNAs in patients with heart disease. Int J Cardiol. 2021;344:149–59. doi: 10.1016/j.ijcard.2021.09.046 34592247

[18] Chaudhari A, Axelsson C, Mattsson Hultén L, Rotter Sopasakis V. Toll-like receptors 1, 3 and 7 activate distinct genetic features of NF-κB signaling and γ-protocadherin expression in human cardiac fibroblasts. Inflammation. 2025;48(5):2896–907. doi: 10.1007/s10753-025-02238-z 39828779 PMC12596343

[19] Gillaux C, Méhats C, Vaiman D, Cabrol D, Breuiller-Fouché M. Functional screening of TLRs in human amniotic epithelial cells. J Immunol. 2011;187(5):2766–74. doi: 10.4049/jimmunol.1100217 21775685

[20] Kang Y, Nii T, Isobe N, Yoshimura Y. Effects of TLR ligands on the expression of cytokines and possible role of NFκB in its process in the theca of chicken follicles. J Poult Sci. 2018;55(4):288–300. doi: 10.2141/jpsa.0170217 32055188 PMC6756407

[21] Fitzner N, Clauberg S, Essmann F, Liebmann J, Kolb-Bachofen V. Human skin endothelial cells can express all 10 TLR genes and respond to respective ligands. Clin Vaccine Immunol. 2008;15(1):138–46. doi: 10.1128/CVI.00257-07 17978010 PMC2223852

[22] Bhagwani A, Thompson AAR, Farkas L. When innate immunity meets angiogenesis-the role of toll-like receptors in endothelial cells and pulmonary hypertension. Front Med (Lausanne). 2020;7:352. doi: 10.3389/fmed.2020.00352 32850883 PMC7410919

[23] Pegu A, Qin S, Fallert Junecko BA, Nisato RE, Pepper MS, Reinhart TA. Human lymphatic endothelial cells express multiple functional TLRs. J Immunol. 2008;180(5):3399–405. doi: 10.4049/jimmunol.180.5.3399 18292566

[24] Biswas I, Singh B, Sharma M, Agrawala PK, Khan GA. Extracellular RNA facilitates hypoxia-induced leukocyte adhesion and infiltration in the lung through TLR3-IFN-γ-STAT1 signaling pathway. Eur J Immunol. 2015;45(11):3158–73. doi: 10.1002/eji.201545597 26350442

[25] Wang X, He B. Endothelial dysfunction: Molecular mechanisms and clinical implications. MedComm (2020). 2024;5(8):e651. doi: 10.1002/mco2.651 39040847 PMC11261813

[26] Ramji DP, Davies TS. Cytokines in atherosclerosis: Key players in all stages of disease and promising therapeutic targets. Cytokine Growth Factor Rev. 2015;26(6):673–85. doi: 10.1016/j.cytogfr.2015.04.003 26005197 PMC4671520

[27] Chi Z, Melendez AJ. Role of cell adhesion molecules and immune-cell migration in the initiation, onset and development of atherosclerosis. Cell Adh Migr. 2007;1(4):171–5. doi: 10.4161/cam.1.4.5321 19262139 PMC2634102

[28] Henein MY, Vancheri S, Longo G, Vancheri F. The role of inflammation in cardiovascular disease. Int J Mol Sci. 2022;23(21):12906. doi: 10.3390/ijms232112906 36361701 PMC9658900

[29] Vanhoutte PM, Shimokawa H, Feletou M, Tang EHC. Endothelial dysfunction and vascular disease - A 30th anniversary update. Acta Physiol (Oxf). 2017;219(1):22–96. doi: 10.1111/apha.12646 26706498

[30] Deravi N, Poudineh M, Pirzadeh M, Yavarpour-Bali H, Mehrabi H, Erabi G, et al. The Yin and Yang of toll-like receptors in endothelial dysfunction. Int Immunopharmacol. 2022;108:108768. doi: 10.1016/j.intimp.2022.108768 35430433

[31] Ando M, Matsumoto T, Taguchi K, Kobayashi T. Poly (I:C) impairs NO donor-induced relaxation by overexposure to NO via the NF-kappa B/iNOS pathway in rat superior mesenteric arteries. Free Radic Biol Med. 2017;112:553–66. doi: 10.1016/j.freeradbiomed.2017.08.027 28870522

[32] Zimmer S, Steinmetz M, Asdonk T, Motz I, Coch C, Hartmann E, et al. Activation of endothelial toll-like receptor 3 impairs endothelial function. Circ Res. 2011;108(11):1358–66. doi: 10.1161/CIRCRESAHA.111.243246 21493895

[33] Tatematsu M, Funami K, Seya T, Matsumoto M. Extracellular RNA sensing by pattern recognition receptors. J Innate Immun. 2018;10(5–6):398–406. 10.1159/000494034 PMC6784046 30404092

[34] Wurfel MM, Gordon AC, Holden TD, Radella F, Strout J, Kajikawa O, et al. Toll-like receptor 1 polymorphisms affect innate immune responses and outcomes in sepsis. Am J Respir Crit Care Med. 2008;178(7):710–20. doi: 10.1164/rccm.200803-462OC 18635889 PMC2556453

[35] Liu J, Jiang M, Ma Z, Dietze KK, Zelinskyy G, Yang D, et al. TLR1/2 ligand-stimulated mouse liver endothelial cells secrete IL-12 and trigger CD8+ T cell immunity in vitro. J Immunol. 2013;191(12):6178–90. doi: 10.4049/jimmunol.1301262 24227786

[36] Johnson RH, Kho DT, O’ Carroll SJ, Angel CE, Graham ES. The functional and inflammatory response of brain endothelial cells to toll-like receptor agonists. Sci Rep. 2018;8(1):10102. doi: 10.1038/s41598-018-28518-3 29973684 PMC6031625

[37] Karadimou G, Folkersen L, Berg M, Perisic L, Discacciati A, Roy J, et al. Low TLR7 gene expression in atherosclerotic plaques is associated with major adverse cardio- and cerebrovascular events. Cardiovasc Res. 2017;113(1):30–9. doi: 10.1093/cvr/cvw231 27864310 PMC5220676

[38] Liu C-L, Santos MM, Fernandes C, Liao M, Iamarene K, Zhang J-Y, et al. Toll-like receptor 7 deficiency protects apolipoprotein E-deficient mice from diet-induced atherosclerosis. Sci Rep. 2017;7(1):847. doi: 10.1038/s41598-017-00977-0 28405010 PMC5429799

